# Education program promoting report of elder abuse by nursing students: a pilot study

**DOI:** 10.1186/s12877-023-03931-0

**Published:** 2023-03-31

**Authors:** Dahye Park, Jeongmin Ha

**Affiliations:** 1 Department of Nursing, Semyung University, 65 Semyung-Ro, Jechoen-Si, Chungbuk, Republic of Korea; 2 Department of Nursing, Dong-A University, 3 Dongdaeshin-Dong Seogu, Busan, 602–714 Republic of Korea

**Keywords:** ADDIE model, Elder abuse, Education program, Nursing students, RE-AIM

## Abstract

**Background:**

Elder abuse is an important public health concern that requires urgent attention. One main barrier to active responses to elder abuse in clinical settings is a low level of relevant knowledge among nurses. This study aims to develop an educational program to promote an intent to report elder abuse among nursing students and assess its effectiveness, with a focus on the rights of older adults.

**Methods:**

A mixed method design was used with the Analyze, Design, Develop, Implement, and Evaluate model. Twenty-five nursing students from Chungbuk Province participated in the study. Attitude toward older adults and knowledge of, awareness of, attitude towards, and intent to report elder abuse were assessed quantitatively and analyzed using paired t-test. The feasibility of the program and feedback were collected qualitatively through group interviews and analyzed using content analysis.

**Results:**

After the education program, attitude toward older adults (Cohen’s d = 1.08), knowledge of (Cohen’s d = 2.15), awareness of (Cohen’s d = 1.56), attitude towards (Cohen’s d = 1.85), and intent to report elder abuse (Cohen’s d = 2.78) increased, confirming the positive effects of this program. Overall, all participants were satisfied with the contents and method of the program.

**Conclusions:**

The method of program delivery should be improved and tailored strategies to boost program engagement among nursing students should be explored to implement and disseminate the program.

## Introduction

Elder abuse is an important public health concern with serious social, economic, and health consequences [1]. In Korea, a 2011 survey of 10,674 older adults aged 65 years and above showed that 12.6%—about one out of every eight older adults—suffered abuse [2]. Moreover, although recent surveys on elder abuse are unavailable, considering the rise in the incidence of elder abuse during the coronavirus disease 2019 (COVID-19) pandemic in the United States [3], the incidence of elder abuse in Korea may have also increased.

In clinical practice, nurses are likely to witness or predict elder abuse as their work involves a careful observation of patients’ daily lives, which provides them with an opportunity to detect, treat, and prevent elder abuse [4]. However, nurses do not actively intervene or connect older adults suffering from abuse to relevant intervention programs, despite having opportunities [5]. This is because of severe barriers to active reporting of elder abuse (e.g., invisibility and caregiver risk factors are common) [6]. Indeed, the number of reports of elder abuse by mandated reporters in 2021 was only 860 out of 7,634—a decline of 8.4% from the 939 reported cases of elder abuse by mandated reporters in 2020 [7].

In most autonomous districts in Korea, visiting nurses provide care to older adults belonging to vulnerable groups in the community [8]. Visiting nurses can determine whether the environment is safe to prevent elder abuse, which is easily concealed in the community, and have the opportunity to detect elder abuse early [9]. However, Korean nurses’ awareness of elder abuse was lower than that of other occupational groups such as nursing care workers and paramedics [9]. As Korean nurses’ awareness of elder abuse was low, there is a great possibility that on witnessing elder abuse while on duty they may not recognize it or cope with it effectively [9].

Nurses’ understanding of elder abuse is an important factor for active responses to elder abuse in a clinical setting. However, nursing students in Korea display poor knowledge on elder abuse [10]. A previous study that investigated Korean nursing students’ elder abuse-related educational needs exploring the difference between the levels of importance and performance using the IPA analysis found that the highest priority knowledge set that must be urgently improved included topics of adults’ physical and emotional changes, sexual abuse, legal punishment for elder abuse, roles of mandated reporters, roles of older adult protection agencies and shelters for elder abuse victims, encouragement of reporting and hotline, and process following abuse reporting [4]. Furthermore, topics comprising the second priority group of knowledge set that must be gradually improved consisted of human rights for older adults, roles of mandated reporters for protecting older adults’ rights, roles for prevention, verbal abuse, physical abuse, emotional abuse, neglect, and abandonment [4].

Data showing that professional knowledge about elder abuse is a potent antecedent to reporting elder abuse [5] highlights the need for a systematic educational program for nursing students in Korea, including expert knowledge about elder abuse, reporting of abuse, and legal and ethical grounds. However, studies that have developed and implemented elder abuse-related educational programs for nursing students in Korea are limited. This study aims to develop an educational program for promoting the intent to report elder abuse among nursing students and assess its effectiveness using the Reach, Efficacy of program under optimal conditions (i.e., intervention study), Adoption, Implementation, Maintenance) framework, an approach frequently mentioned in implementation and dissemination research [11–13]. We sought to assess the first two out of five elements of the framework: (1) to promote intent to report elder abuse incidents and to investigate the effects of the education program on attitude toward older adults, knowledge of, awareness of, attitude towards, and intent to report elder abuse; (2) to collect feedback from users through group interviews and analyze the feedback using content analysis to improve the feasibility of the program.

## Method

### Study design

**Fig. 1:**
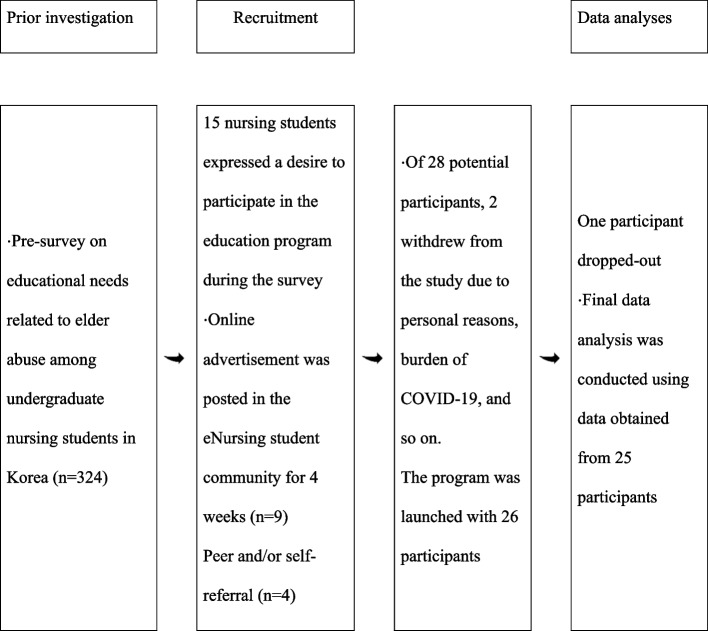
The process of participant recruitment

This pilot study aims to develop an education program for promoting intent to report elder abuse incidents among nursing students and to examine the feasibility of the program nationwide. After developing the program, we used a mixed method design to collect baseline data to pivot the program for effective adoption in practice (Fig. 1). We used one group pretest–posttest design in this pilot study to develop and assess the educational program, aiming to improve intent to report elder abuse among nursing students.

### Study participants

Nursing students were set as the study population and the only eligibility criterion was the ability to participate in four sessions of education provided on an offline platform over two months. There were no other criteria, including age and gender. There are no established standards for sample determination for pilot studies. Thus, we determined our sample size with reference to the previous findings that suggested the optimal sample size to be 10–30 participants with the same characteristics as the targeted study population [11, 14]. In consideration of four sessions of education over a period of two months, we recruited 28 nursing students for this pilot study.

Figure 1 shows the participant recruitment process. First, in the preliminary study on the educational needs for elder abuse among nursing students, we advertised the elder abuse-related education program and asked students who were interested to leave their phone numbers. Of 324 survey respondents, 15 showed interest in the program and left their phone numbers.

Next, we posted an advertisement on an online bulletin board for nursing students for four weeks from September 3, 2020, to October 3, 2020, and nine nursing students showed interest in participating in the study. Four students were additionally recruited through peer and self-referral. However, 2 of 28 nursing students who showed interest in the study withdrew their decision to participate in the study during the informed consent process either for personal reasons or due to COVID-19 restrictions.

### Ethical consideration

This study was approved by the Institutional Review Board at S University (IRB No S**-2020–08-011). The participants were informed about the purpose of the study, voluntary participation, freedom to withdraw from the study, guarantee of anonymity, and use of collected data for only research purposes, and written consent was obtained after confirming that the participants have accurately understood the purpose, procedure, and method of the study using the talk-back method.

### Study procedure

#### Development of education program

The education program used as the intervention in this study was developed based on the Analyze, Design, Develop, Implement, and Evaluate model, a widely used generic model for instructional design [15]. This model comprises five steps: analysis, designing, development, implementation, evaluation as shown in Table 1.

**Table 1:** Developing an educational program for promoting nursing students’ intention to report elder abuse using the Analyze, Design, Develop, Implement, and Evaluate model

Step	Process	Educational program for promoting nursing students’ intention to report elder abuse
Analysis	1. Topic analysis	1. Review literature
	2. Learner analysis	2. Check course subject
	3. Learner needs analysis	3. Needs assessment: survey
Design	1. Set learning objectives	1. Set educational goals
	2. Select learning contents	2. Select educational contents and topics
	3. Select learning strategy	3. Choose educational media and methods
Development	1. Development of educational materials	1. Development of PPT of educational program and selection of a case
	2. Validation of content	2. Validity of content valuation by experts
	3. Final program development	3. Complement modification
Implementation	1. Preparation of the program	1. Preliminary survey & complement modification
	2. Application of the program	2. Launching the program
Evaluation	1. Evaluation of the educational program	1. Evaluation of the educational program by learners

#### Implementation of the education program

A 240-min offline education program developed by the researchers for nursing students was provided over four Saturday sessions. The details of the program are shown in Table 2. This program’s design differed from existing programs as it not only comprised frontal teaching, but also more participative methods, such as brain writing. Brain writing, a teaching and learning method known to effectively collect ideas and solve problems, was applied. The contents included in this program were: understanding of older adults’ rights and abuse, definition and types of elder abuse, current status and laws pertinent to elder abuse, professional older adult protective agencies, and tips for reporting elder abuse.

**Table 2:** Educational program for promoting nursing students’ intention to report elder abuse

Category	Contents	Operating time	Instructor
Understanding of older adults rights and abuse	1. Understanding of human rights of older adults 1) Human rights 2) UN Principles for older adults	15 min	Research team
	2. Current status of human rights for older adults	15 min	
	3. Roles of mandated reporters to protect the human rights of older adults	30 min	
Definitions and types of elder abuse	1. Definitions and types of elder abuse 1) Definition of elder abuse 2) Types of elder abuse 3) Characteristics of elder abuse 4) Elder abuse victims and characteristics of perpetrators	30 min	Research team
	2. Cases of elder abuse 1) Physical abuse 2) Emotional abuse 3) Sexual abuse 4) Financial abuse 5) Neglect 6) Abandonment	30 min	Research team
Current status and laws pertaining to elder abuse	1. Current status of elder abuse 1) Number of reported elder abuse cases 2) Elder abuse by type of incident 3) Perpetrators 4) Institutional abuse	20 min	Research team
	2. Understanding laws pertaining to elder abuse 1) Laws for punishment for elder abuse 2) Laws for mandated reporters 3) Laws for intervention in elder abuse	40 min	Research team
Older adult protective agencies and methods of reporting	1. Utilizing older adult protective agencies and shelters for elder abuse victims 1) Older adult protective agencies 2) Shelters for elder abuse victims	30 min	Research team
	2. Methods of reporting 1) Process of intervening in an elder abuse case 2) Roles of mandated reporters 3) Reporting elder abuse, training, and debriefing	30 min	Research team

#### Instruments

Before beginning offline education, we administered a survey to examine participants’ attitudes toward older adults, knowledge of, awareness of, attitude towards, and intent to report elder abuse. The same survey was administered immediately after the program was completed. Furthermore, group interviews were conducted using semi-structured questions after education to obtain feedback about the program and improve its feasibility.

#### Attitude toward older adults

Attitude toward older adults was measured using the Semantic Differential Scaling developed by Sanders et al. [16] and adapted by Im [17]. This instrument comprises 20 pairs of contradictory adjectives, and each adjective pair is rated on a seven-point Likert scale (1 = very negative, 7 = very positive). In a previous study, seven items (#1, 2, 3, 6, 7, 10, 15, 18) were reverse coded to maintain consistency in scoring. The average score of 20 items was used; a higher score indicated a positive attitude toward older adults, with a score of 3.5–4.5 indicating a neutral attitude [16]. The reliability (Cronbach's α) of the scale was 0.90 at the time of development, 0.82 in the study by Im [17], 0.80 at the baseline, and 0.82 at the post-test in this study.

#### Knowledge about elder abuse reporting

Knowledge about elder abuse reporting was measured using a 12-item questionnaire. The first author developed this questionnaire based on previous studies and the Welfare of the Senior Citizens Act, which includes the regulations for punishment for elder abuse (Article 55–2, 3, 4; Article 57), duty and procedure of elder abuse reporting (Article 39–6), and older adult protective agencies (Article 39–5). The questionnaire comprised two items for definition (concept and type), five items for law (mandated reporter and organizations), and five items for system (reporting organization and process). Participants were asked to check “I don’t know” or “I am well aware of it,” which were scored as 0 and 1, respectively. The total possible score for knowledge about elder abuse reporting ranged from 0–12, and a higher score indicated a greater level of knowledge. The Cronbach’s α was 0.77 at the baseline and 0.82 at the post-test in this study.

#### Awareness of elder abuse

Awareness of elder abuse offences was measured using 12 scenarios developed by Moon and Williams [18] and translated and adapted in Korea by Yoo and Kim [19]. The 12 scenarios are divided into five domains: physical abuse, emotional abuse, financial abuse, sexual abuse, and neglect. Specifically, there were three physical abuse scenarios (scenarios 1, 3, 4), four emotional abuse scenarios (scenarios 2, 5, 6, 10), two financial abuse scenarios (scenarios 8, 11), two neglect scenarios (scenarios 7, 9), and one sexual abuse scenario (scenario 12). Each scenario was rated on a five-point Likert scale from 1 “This is not an abuse” to 5 “This is a very serious abuse.” The mean overall score and scores by domain were used. The total score ranged from 12–60, and a higher score indicated greater awareness of elder abuse. The reliability (Cronbach’s α) score was 0.77 in the study by Yoo and Kim [19], 0.66 at the baseline, and 0.72 at the post-test in this study.

#### Attitude toward elder abuse

Attitude toward elder abuse was measured using the tool developed for older adults by Cho [20]. This 25-item tool comprises fourteen items for attitude, four items for subjective norms, and seven items for perceived behavioral control. Each item is rated on a four-point Likert scale (1 “strongly disagree,” 2 “disagree,” 3 “agree,” 4 “strongly agree”), with a higher score indicating more positive attitude toward intervening in the situation. Some examples of the items for attitude include “If I report an elder abuse incident, the organization that receives the report will take necessary actions,” and “Intervening in elder abuse will be helpful for the older adult involved.” There were four items for subjective norms, but two items pertinent to coworkers and head nurse were deleted because our participants were students. The scores rated on a four-point scale were summed. Cronbach’s α of the scale was 0.77 for attitude, 0.74 for subjective norms, and 0.73 for perceived behavioral control in a previous study, and 0.72 for the baseline and 0.84 for the post-test in this study.

#### Intent to report

Intent to report was assessed by having the participants answer yes (1) or no (0) to the question asking whether they will report each of the 12 hypothetical scenarios presented earlier. The total score was calculated by summing the score for 12 scenarios [18]. Cronbach’s α was 0.64 at the baseline and 0.68 at the post-test in this study.

#### Group interview to obtain feedback for elder abuse education program

At the end of the program, a group interview was conducted using semi-structured questions to collect feedback on the 4-week program. The three semi-structured questions used were: “What motivated you to participate in the program?”; “What were some of the positive experiences and difficulties you faced while participating in the program?”; “What should the researchers consider when revising the education program for nursing students?”.

### Data collection and analysis

The data were collected between September 3, 2020, and April 31, 2021. Of the 28 nursing students who showed interest to participate in the study, 26 were enrolled in the study, and 25 out of the 26 completed the program. Quantitative data collected from one student who withdrew in the middle of the program was excluded from the analysis, so data from 25 participants were included in the final quantitative analysis. Quantitative analysis was performed using SPSS 25.0 software, and the reliability of the instruments, frequency, and descriptive statistics were analyzed. The differences in the scores before and after education were compared using paired t-test. The effect size was calculated using Cohen’s d due to the small sample size. The normality of the data was tested using the Shapiro–Wilk test, and normal distributions of attitude toward older adults, knowledge of, awareness of, attitude towards, and intent to report elder abuse were checked.

The interview was conducted by the first author, who had experience in qualitative research. The researcher used a list of semi-structured questions and audio-recorded the interviews. Further, an assistant researcher observed participants’ reactions and took field notes as necessary. The interviews were transcribed, and the first author and another author independently performed content analysis to extract themes by category, theme clusters, and categorization.

## Results

### Participants’ sociodemographic characteristics

Table 3 shows the participants’ sociodemographic characteristics. The majority of participants were women (88.0%), and the mean age was 21.8 years, ranging from 20–28 years. Only 10 participants had prior education about elder abuse or exposure to an elder abuse awareness campaign before enrolling in the study. Regarding intervening in an elder abuse case, 12 participants stated that they would only report the incident before the education, while 4 stated that they would do so after the program. Moreover, before the education, 2 participants indicated that they would report the incident and intervene with only the older adult, while after the program, 10 stated that they would do the same.

**Table 3:** General characteristics

Item	Frequency (%)	
	Pre	Post
Gender		
Male	3(12.0)	3(12.0)
Female	22(88.0)	22(88.0)
Age (year) Average age = 21.84 ± 1.72		
20–21	11(44.0)	11(44.0)
22–23	10(40.0)	10(40.0)
24-	4(16.0)	4(16.0)
Religion		
Christian	8(32.0)	15(60.0)
Catholic	2(8.0)	8(32.0)
none	15(60.0)	2(8.0)
Education		
0	15(60.0)	0(0.0)
1	10(40.0)	15(100.0)
Intervention		
Only report	12(48.0)	4(16.0)
Report, and intervene with family only	1(4.0)	0(0.0)
Report, and intervene with older adult only	2(8.0)	10(40.0)
Report, and intervene with both older adult and family	10(40.0)	11(44.0)

### Effects of the education program on intent to report elder abuse among nursing students

After the program, nursing students’ attitude toward older adults (Cohen’s d = 1.08), knowledge (Cohen’s d = 2.15), awareness (Cohen’s d = 1.56), attitude (Cohen’s d = 1.85), and intent to report elder abuse (Cohen’s d = 2.78) increased, confirming the positive effects of the program.

Table 4 shows the results before and after the education.

**Table 4:** Changes in attitude toward older adults, knowledge and awareness of, attitude toward, and intent to report elder abuse after the education program (*n* = 70)

Variable	Pre	Post	t	p	Cohen's *d*
Attitude toward older adults	80.88 ± 11.39	92.4 ± 9.84	-6.38	.028	1.08
Knowledge about elder abuse	4.76 ± 1.79	8.84 ± 1.99	-12.13	< .001	2.15
Awareness of elder abuse	45.16 ± 5.38	52.32 ± 3.57	-8.75	< .001	1.56
Physical abuse	11.72 ± 1.57	13.40 ± 1.50	-5.25	< .001	1.09
Emotional abuse	11.76 ± 3.57	15.32 ± 2.32	-7.21	< .001	1.18
Financial abuse	8.00 ± 1.32	8.88 ± 1.05	-2.37	.026	0.73
Neglect	8.80 ± 1.19	9.72 ± 0.46	-4.13	< .001	1.01
Sexual abuse	4.88 ± 0.33	5.00 ± 0.00	-1.81	.083	0.51
Attitude toward elder abuse	56.16 ± 3.90	63.12 ± 3.61	-7.41	< .001	1.85
Attitude	32.80 ± 3.07	35.88 ± 2.77	-5.23	< .001	1.05
Subjective norm	5.32 ± 1.21	6.56 ± 1.00	-4.55	< .001	1.11
Perceived behavioral control	18.04 ± 2.75	20.68 ± 2.50	-3.27	.003	1.00
Intent to report elder abuse	32.08 ± 1.73	39.76 ± 3.50	-9.21	< .001	2.78

Attitude toward older adults significantly increased from 80.88 ± 11.39 at the baseline to 92.4 ± 9.84 after education (t = -6.38, p = 0.028, Cohen’s d = 1.08). Knowledge about elder abuse significantly increased from 4.76 ± 1.79 at the baseline to 8.84 ± 1.99 after education (t = -12.13, *p* < 0.001, Cohen’s d = 2.15). Awareness of elder abuse significantly increased from 45.16 ± 5.38 at the baseline to 52.32 ± 3.57 after education (t = -8.75, *p* < 0.001, Cohen’s d = 1.56). By domain, the scores for physical abuse (t = -5.25, *p* < 0.001), emotional abuse (t = -7.21, *p* < 0.001), financial abuse (t = -2.37, *p* = 0.026), and neglect (t = -4.13, *p* < 0.001) statistically significantly increased.

Attitude toward elder abuse significantly increased from 56.16 ± 3.90 at the baseline to 63.12 ± 3.61 after education (t = -7.41, *p* < 0.001, Cohen’s d = 1.85). By domain, the scores for attitude (t = -5.23, *p* < 0.001), subjective norms (t = -4.55, *p* < 0.001), and perceived behavioral control (t = -3.27, p = 0.003) statistically significantly increased.

Intent to report elder abuse significantly increased from 32.08 ± 1.73 at the baseline to 39.76 ± 3.50 after education (t = -9.21, *p* < 0.001, Cohen’s d = 1.08).

## Participants’ feedback on education program for promoting nursing students’ intent to report elder abuse

### Reason for participating in the program

The reasons for participating in the program included self-improvement (*n* = 2), increased perceived need for education after the informed consent process (*n* = 1), exploring topics for course assignment (*n* = 1), perceived need for education during practicum (*n* = 5), perceived need for education while providing care for families of older adults (*n* = 1), and had an interest in the topic (*n* = 1). The participants were satisfied with the education program overall.

### Benefits and challenges of the program

#### Learning about care for older adults

Students mentioned learning about different types of elder abuse and laws pertaining to elder abuse as a benefit of participating in the program. Furthermore, they also stated that they can utilize what they have learned to provide more meaningful care for older adults.

Developing competencies as a nursing student (formal Para).

Most students stated that they liked the fact that the education program was free and that they participated to learn instead of just passing an exam. Unlike other education programs, they were allowed to relieve their skepticism about nursing by interacting with their fellow students proudly about nursing and engaging in introspection, based on which they were able to better recognize the value of nursing.

#### Feedback for improving the feasibility of the program

##### Need to emphasize that the program is about reporting elder abuse

Many nursing students who saw the advertisement poster misunderstood the program as a geriatric nursing program when they were recruited. Thus, the students advised that we emphasize the program as an educational program for nursing students whose prospective mandates reporting elder abuse.

##### Development of an e-learning program

With in-person activities restricted during the COVID-19 pandemic, students suggested developing strategies that allow more nursing students to access the program. For example, they recommended converting the offline program to an e-learning program for promoting intent to report elder abuse.

## Discussion

This pilot study aimed to develop an educational program to promote intent to report elder abuse among nursing students and assess its effectiveness. Additionally, we aimed to collect feedback from participants to improve the feasibility of the program. After administering the developed education program about elder abuse, students showed improved attitudes toward older adults, knowledge of, awareness of, attitude towards, and intent to report elder abuse, confirming the positive effects of the program.

These results support the findings of a previous study that investigated awareness, subjective norms, perceived behavioral control, and attitude toward elder abuse among nursing students in Korea, where students who took a relevant course demonstrated a higher level of awareness, subjective norms, and perceived behavioral control [10]. Therefore, continuously providing a systematic education program on elder abuse reporting to nursing students, especially during a time witnessing various instances of elder abuse due to the burgeoning older adult population [21] and Korean nurses’ awareness of elder abuse being low compared to other occupational groups in Korea [9], will not only bolster nursing students’ competencies for responding to elder abuse incidents but will also contribute to addressing the crucial societal issue. However, it is necessary to update how the program is promoted and modify the current program, particularly in light of comments that various platforms of operation should be explored to facilitate the implementation and dissemination of the program. Therefore, we will discuss three factors that should be improved.

First, the title of the program should be chosen such that the contents of the program are clearly conveyed when advertising the program. The objective of the education program developed in this study was to educate nursing students about their duty to report elder abuse. However, some participants misunderstood the program as an educational program for geriatric nursing during the recruitment process. Further, none of the participants in the program demonstrated an interest in elder abuse reporting or an increased desire to improve their ability to report the same. Therefore, the title of the program should reflect nursing students’ opinions. Students’ opinions can be collected by asking them to choose from a list of a few titles. Further, considering that a contest enhances the credibility of a company and boosts individuals’ willingness to be involved in the said company [22], launching a naming contest for the program could attract students’ attention, deepen their understanding of the program, and increase the participation rate.

Second, the instructors need to use questions that trigger thinking to give students adequate opportunities to think about the topic (reporting elder abuse) on their own and discuss it among them. The students reported that they experienced a positive emotional process where they felt more value in nursing as they contemplated about a topic with fellow students and engaged in introspection and reflection. Nurses in geriatric hospitals in Korea experience ethical conflicts as “being distressed,” namely moral distress, which refers to being unable to do the right thing despite being aware of it [23]. One main cause of nurses’ distress is a working environment that does not fulfill ethical obligations [23]. In East Asian contexts, elder abuse is pervasively perceived as a personal family issue. Family matters and issues are kept within the family, as sharing them with outsiders can expose the family to public embarrassment and lead to loss of face. Besides, other reasons why older adults remain silent include a culture‐specific misunderstanding of elder abuse, shame, self‐blame, and the belief of inescapable ill fate [24].

Therefore, mandated reporters in Korea choose not to execute mandatory reporting because they feel that by modifying the conditions that cause abuse, family members can participate in providing care for older adults at home. They believe that providing care at home, improving the relationship between older adults and their families, and intermediation provides a better cultural option for older adults [25]. Additionally, older Korean adults, as victims, expressed reluctance to seek help or attention despite abuse experiences due to a culture of family honor and filial piety, an obligation to uphold norms, such as endurance and self-effacement, and belief in fatalism (acceptance of fate) [24]. To address this problem, education programs using verified teaching methods, such as the nudge strategy, and exemplary cases that arouse moral emotions advancing from the conventional moral education focused on character and virtue towards a cognitive approach should be actively developed [26].

Third, a non-face-to-face education program should be developed. We could not expect students’ active participation amid restrictions on in-person activities due to the COVID-19 pandemic. The students also suggested that a non-face-to-face education program should be developed. In a recent study, nurses and social workers who participated in virtual-reality-based elder abuse and neglect educational intervention acquired knowledge about identifying elder abuse and neglect and demonstrated 99% accuracy in their decisions for mandatory reporting. Further, the knowledge and skills they acquired in the intervention brought positive changes in their actual work performance [27]. These results suggest that a non-face-to-face education program can adequately alter knowledge and teaching skills that may have a positive impact in a clinical setting. Therefore, developing a non-face-to-face version of our program would provide effective education for a larger population of nursing students.

## Conclusion

This pilot study aims to develop an educational program to promote the intent to report elder abuse among nursing students and assess its effectiveness. Despite the strength—developing and examining the effectiveness of an education program about elder abuse reporting based on nursing students’ educational needs—this study has a few limitations. First, some participants misunderstood this program as an education program for geriatric nursing instead of elder abuse. This misunderstanding could have contributed to the lower scores found on the pre-test. Thus, the educational goal should be clarified and promoted in the process of participant recruitment to confirm this program’s effectiveness in the follow-up study. Second, all participants were students at a single school. Additionally, students volunteered to participate in this offline program while all other courses were administered online, which indicates that only students with high educational needs may have been recruited. Thus, our findings cannot be generalized. Studies including nursing students from various regions and diverse demographic backgrounds are needed. In summary, future research should focus on planning preventive measures for elder abuse, developing suitable training programs, and supporting the older adult population. The method of program delivery should be improved and tailored strategies to boost program engagement among nursing students should be explored to implement and disseminate the program.

## Data Availability

All data generated or analysed during this study are included in this published article.
